# Auditory evoked magnetic fields in individuals with tinnitus^☆^

**DOI:** 10.1016/j.heares.2013.04.006

**Published:** 2013-08

**Authors:** Magdalena Sereda, Peyman Adjamian, Mark Edmondson-Jones, Alan R. Palmer, Deborah A. Hall

**Affiliations:** a National Institute for Health Research Nottingham Hearing Biomedical Research Unit, School of Clinical Sciences, University of Nottingham, Ropewalk House, 113 The Ropewalk, NG1 5DU, Nottingham, UK; b MRC Institute of Hearing Research, University Park, Science Road, NG7 2RD, Nottingham, UK

**Keywords:** ABR, auditory brainstem responses, ANOVA, analysis of variance, EEG, electroencephalography, HL, hearing loss, MEG, magnetoencephalography, PTA, pure tone average, SL, sensation level, TI, tinnitus, THI, Tinnitus Handicap Inventory

## Abstract

Some forms of tinnitus are likely to be perceptual consequences of altered neural activity in the central auditory system triggered by damage to the auditory periphery. Animal studies report changes in the evoked responses after noise exposure or ototoxic drugs in inferior colliculus and auditory cortex. However, human electrophysiological evidence is rather equivocal: increased, reduced or no difference in N1/N1m evoked amplitudes and latencies in tinnitus participants have been reported.

The present study used magnetoencephalography to seek evidence for altered evoked responses in people with tinnitus compared to controls (hearing loss matched and normal hearing) in four different stimulus categories (a control tone, a tone corresponding to the audiometric edge, to the dominant tinnitus pitch and a tone within the area of hearing loss). Results revealed that amplitudes of the evoked responses differed depending on the tone category. N1m amplitude to the dominant tinnitus pitch and the frequency within the area of hearing loss were reduced compared to the other two categories. Given that tinnitus pitch is typically within the area of hearing loss, the differences in the evoked responses pattern in tinnitus participants seem to be related more to the hearing loss than to the presence of tinnitus.

## Highlights

• We use MEG to compare evoked responses in participants with tinnitus and controls.
• We compare N1m responses to control, edge, tinnitus and hearing loss tone.
• N1m amplitudes to the tinnitus and hearing loss tones are reduced.
• Differences in N1m amplitudes are related to hearing loss rather than tinnitus.
• There are no tinnitus-specific changes in the pattern of N1m responses.

## 1 Introduction

Recent theories postulate that some forms of tinnitus are a perceptual consequence of altered neural activity in the central auditory system triggered by damage to the auditory periphery whereby the abnormal activity along the auditory pathway is erroneously interpreted as a sound (Eggermont and Roberts, 2004). While loss of afferents following cochlear damage may be the initiating cause in the peripheral system, central mechanisms are probably crucial for maintaining tinnitus. Animal studies have provided a wealth of evidence in favour of this view and have been the main source of our current knowledge regarding neurophysiological correlates of tinnitus (for a recent review see Noreña, 2011). In addition to reduced output from the cochlea and auditory nerve after noise exposure or ototoxic drugs, many animal studies have observed concomitant changes in the ascending auditory pathway both at rest (spontaneous activity) and in response to external sounds (evoked activity). Following sensory deafferentation after noise trauma or ototoxic drugs neurons within the central auditory system can become hyperexcitable. For example, at the level of auditory cortex increased sound-evoked firing rate has been shown after noise exposure in cats (Kimura and Eggermont, 1999; Noreña et al., 2003) and those activity changes appeared to be greatest for frequencies below the hearing loss (Noreña et al., 2003). The amplitude of the sound-evoked response has generally been shown to increase after noise exposure in the auditory cortex of chinchillas (Salvi et al., 2000), cats (Noreña et al., 2003), guinea pigs (Popelar et al., 1987; Syka et al., 1994) and rats (Syka and Rybalko, 2000; Popelar et al., 2008; Sun et al., 2008).

Several mechanisms have been proposed to explain changes in the evoked responses associated with tinnitus. One view proposes an unmasking of the excitatory activity due to loss of lateral inhibition as a result of hearing loss (Gerken, 1996). A second pervasive model gives primacy to the notion that cochlear damage results in the reorganisation of the tonotopic map in central auditory structures so that neurons at the audiometric edge of the hearing loss acquire the characteristic frequency of their unaffected neighbouring areas. As a consequence, more neurons respond to the frequency at the edge and a resulting enhancement of the evoked response to that frequency can be observed (Robertson and Irvine, 1989; Rajan et al., 1993). More recent models postulate increased spontaneous neuronal activity corresponding to the hearing loss region through homeostatic plasticity (Noreña, 2011; Schaette and Kempter, 2006). Homeostatic plasticity is postulated to stabilise the neural activity in cases of auditory deprivation by scaling up the strength of excitatory synapses and scaling down the strength of inhibitory synapses, which results in increased excitability of neurons. Empirical evidence for this is lacking in humans, but recent data using auditory brainstem responses (ABR), showed reduction of wave I and normalisation of wave V in participants with tinnitus and normal hearing in comparison to normal-hearing controls (Schaette and McAlpine, 2011).

Whatever the precise neural mechanism for tinnitus, the implication for human neuroimaging studies using electroencephalography (EEG) or magnetoencephalography (MEG) is that an increase in neural excitability probably elevates the amplitude of the sound-evoked response, and possibly also affects its latency. Moreover, we might expect differences in the amplitude and/or latency of the evoked responses to predominantly affect either the edge frequency of the hearing loss and/or a frequency corresponding to the dominant tinnitus pitch relative to a control tone that falls within the region of normal hearing. The N1 (EEG) or N1m (MEG) component of the auditory evoked response would seem relevant for studying tinnitus-related activity because it is a reliable cortical response that reflects stimulus properties such as frequency (Näätänen and Picton, 1987). The characteristics of the N1/N1m are also purported to reflect auditory selective attention (Näätänen and Picton, 1987), which is also thought to play a role in tinnitus (e.g., Gu et al., 2010; Hallam et al., 2004). In normal-hearing people, the N1 amplitude typically decreases as a function of frequency (Naka et al., 1999; Fujioka et al., 2002; Gabriel et al., 2004) while changes in latency are usually less pronounced and different studies report mixed results (Roberts and Poeppel, 1996; Naka et al., 1999; Gabriel et al., 2004).

Previous studies have investigated the N1/N1m response as a correlate of tinnitus, but these have yielded rather inconsistent results. A number of studies have predicted generalised hyperexcitability, testing this question by measuring activity evoked by a low-frequency tone (often 1 kHz) corresponding to the region of normal hearing thresholds. Hoke et al. (1989, 1998) used MEG to demonstrate enhanced amplitude of N1m response in people with lateralised tinnitus and hearing loss compared to normally hearing controls, with no difference in N1m latency. Weisz et al. (2005) reported similar group amplitude differences for a low-frequency tone (one octave below the audiometric edge frequency), but this difference was limited to the right hemisphere only. The case study by Pantev et al. (1989) is broadly consistent with these results. As the patient recovered from tinnitus over an 8-month period, N1m decreased in amplitude, while latency remained unchanged. In contrast, Attias et al. (1993) found reduced N1 amplitude for the tinnitus group compared to hearing-matched controls using EEG. Similarly, Jacobson and McCaslin (2003) showed that people with tinnitus demonstrated significantly smaller N1 amplitudes for 0.5 and 1 kHz tones than normally hearing controls, despite these frequencies being in the region of normal auditory sensitivity for both groups. While neither of these EEG studies reported a group difference in N1 latency, Noreña et al. (1999) found that N1 latencies in participants with bilateral tinnitus were shorter than those in hearing-matched controls, but only at the highest sound intensities (80 and 90 dB SPL). However, it is difficult to draw any conclusions about absolute amplitude of N1 component alone because Noreña et al. (1999) report only the difference between N1 and P2 components. Several MEG studies using 1-kHz tones have failed to find any systematic differences between N1m for people with tinnitus and normally hearing controls in either evoked amplitude or latency (Jacobson et al., 1991; Colding-Jorgensen et al., 1992).

A prediction from the viewpoint of tonotopic reorganisation is that there should be an enhanced response to a frequency corresponding to the audiometric edge of a sloping high-frequency hearing loss. One MEG study found a significant increase of cortical strength values (dipole moments) for the audiometric edge frequency compared to lower frequencies in people with hearing loss, with seven out of eight of these also experiencing tinnitus (Dietrich et al., 2001). These authors postulate that this effect is due to expansion of the cortical representation of the edge frequency. However, it is uncertain whether this frequency-specific effect is a marker for tinnitus because a subsequent study found no between-group differences in N1m dipole strength or N1m latency for a frequency corresponding to the audiometric edge (tinnitus with hearing loss versus normally hearing controls, Weisz et al., 2005).

Other studies have sought evidence for enhanced responses corresponding to the tinnitus frequency, which is often within the region of hearing loss, by investigating intensity dependence of a tone corresponding to the dominant tinnitus pitch (Kadner et al., 2002; Pineda et al., 2008). They postulated that tinnitus-related activity would produce an increase in neuronal firing rate or activation of a greater neural substrate, which would result in enhanced intensity dependence of the responses to tones at the tinnitus frequency. Kadner et al. (2002) found that, in tinnitus subjects with hearing loss, responses to the tinnitus frequency were slightly more intensity dependent, with steeper intensity response curves, while responses to 2-kHz tones (approximately one octave below the tinnitus frequency) were slightly less intensity dependent than in normally hearing controls (less steep intensity response curve). The authors suggested that this is due to lateral inhibition caused by tinnitus-related activity. In agreement with the above study, Pineda et al. (2008) demonstrated decreased intensity dependence of responses to the tinnitus frequency after three weeks of customised sound therapy in tinnitus patients, making these responses more similar to controls. They proposed that higher slopes of intensity functions in tinnitus patients indicate reorganisation of the cortical tonotopic map, which might be reversed with customised sound therapy.

In addition to the N1m, some of the above MEG studies have also analysed the P2m response (Hoke et al., 1989, 1998; Jacobson et al., 1991; Colding-Jorgensen et al., 1992; Noreña et al., 1999). Some of these studies that found differences between tinnitus participants and controls with a reduced P2/P2m component in tinnitus subjects (Hoke et al., 1989, 1998; Attias et al., 1993). However, it is noteworthy that Jacobson et al. (1991) reported that the P2m component was often absent in control participants. In most individuals (22 out of 25) P2m was reduced and this resulted in a P2m/N1m ratio below the 0.5 value that Hoke et al. (1989) used as a lower limit of their objective classification criterion for having tinnitus. It is possible that the orientation of the P2m generators relative to the MEG sensors render the imaging technique rather weakly sensitive for detecting this component of the evoked signal. Indeed, EEG seems more sensitive than MEG in detecting the P2 component as all participants in the above study demonstrated normal P2 in EEG recordings (Jacobson et al., 1991). For this reason, the present study assessed the N1m component alone; the most reliable evoked component to be seen in individual listeners.

There are several major challenges to consolidating the outcomes from the different studies. First, authors chose to present different stimulating tones; corresponding to a normal hearing frequency, the audiometric edge or a frequency corresponding to the dominant tinnitus pitch. Moreover, a tone in the low-frequency range (typically 1 kHz) is a somewhat inappropriate stimulation paradigm for directly addressing questions about neuroplasticity in the deafferented frequency region. To the best of our knowledge, no study to-date has assessed responses across frequency categories and yet such an experimental design could be highly informative. Second, some authors chose to compare their tinnitus group with normally hearing controls, while others used controls matched for hearing loss. Many studies do not fully report data on audiometric profile (e.g. Hoke et al., 1989) or simply average evoked responses from participants with varying degrees of hearing loss (e.g. Attias et al., 1993). In order to be able to attribute any abnormal pattern of neural activity to tinnitus *per se*, separate from hearing loss, we and others have previously argued that it is important to closely match tinnitus individuals and controls for hearing loss (e.g. Melcher et al., 2000; Adjamian et al., 2009; Lanting et al., 2009). Indeed, when we applied an audiometric matching procedure in a study measuring the resting-state oscillatory brain rhythms associated with tinnitus, we observed hearing loss to have important interactive effects with tinnitus (Adjamian et al., 2012). On the whole, previous neuroimaging studies have rarely screened participants for other co-morbid conditions which might also be associated with changes in neural firing. Reduced sound level tolerance (hyperacusis) is one such condition (Vernon, 1987) that has been linked with increased neural excitability at multiple levels of the ascending auditory pathway (e.g. Gu et al., 2010). Again, recent reviews have called for screening for hyperacusis in neuroimaging studies of tinnitus (e.g. Adjamian et al., 2009; Lanting et al., 2009).

In this study, we used MEG to investigate the amplitude and latency of evoked magnetic fields in response to a range of audible frequencies in participants with and without tinnitus. We tested the hypothesis that tinnitus-related activity enhancement is evident in the amplitude of the N1m response, even after accounting for explanations in terms of hearing loss and hyperacusis. We also investigated whether any such abnormal response patterns are frequency dependent. To separate the effect of reorganisation due to prolonged tinnitus from that due to sensorineural hearing loss, our study recruited two control groups; one with normal hearing and no tinnitus and the other with matched hearing loss, but without tinnitus.

## 2 Methods

### 2.1 Participants and screening tests

For the MEG study we recruited 22 participants experiencing tinnitus (11 males, 11 females; mean age 55 years 8 months) and 20 controls (8 males, 12 females; mean age 50 years 5 months; Table 1). Participants with tinnitus and with hearing loss were recruited from Nottingham Ear, Nose and Throat (ENT) clinic and Nottingham Audiology Services. A screening questionnaire ensured that all tinnitus participants had experienced the condition for at least 6 months prior to recruitment. Control participants with no tinnitus or hearing loss were recruited from the general public.

Overall, 29 participants were excluded. Twenty-seven were excluded for one of the following reasons: objective or intermittent tinnitus, neurological disorders, current drug prescriptions that could influence the central nervous system, metal implants or devices in the body and stapedectomy surgery for middle ear conditions. Two further participants were excluded due to a diagnosis of hyperacusis (i.e. ≥ 28 on the hyperacusis questionnaire; Khalfa et al., 2002). Three participants were recruited, but withdrew prior to MEG scanning. These individuals are not reported in Table 1. All included participants were right-handed as assessed by Edinburgh Handedness Inventory (Oldfield, 1971). Audiometric thresholds for left and right ears were measured from 0.5 to 12 kHz using standard audiometric procedures. Tinnitus was defined on the basis of self-report and severity of the symptoms was then assessed by the Tinnitus Handicap Inventory (THI, Newman et al., 1996, Table 1).

Of the 22 people with tinnitus, most had a high-frequency hearing loss (*N* = 17). Five had normal hearing defined as pure tone average (PTA) for the frequencies between 0.25 and 8 kHz≤20. However, four of these had some degree of high-frequency hearing loss above 8 kHz. Three had an asymmetric hearing loss defined as a pure-tone average (0.5, 1, 2, and 3 kHz) difference of ≥15 dB (The Food and Drug Administration guidelines). One of the participants had low-frequency hearing loss with normal thresholds from 4 kHz.

Individual psychoacoustic properties of the tinnitus percept were assessed using the Tinnitus Tester (Roberts et al., 2006, 2008). Thus we acquired measures of tinnitus laterality, loudness, categorisation of its spectral properties (tonal, ringing and hissing) and its frequency spectrum (likeness ratings across frequencies, 0.5–12 kHz) (see Table 1). Dominant tinnitus pitch was taken from the pitch-similarity ratings and was defined as the frequency that was rated as the most similar to the tinnitus pitch.

**Fig. 1:**
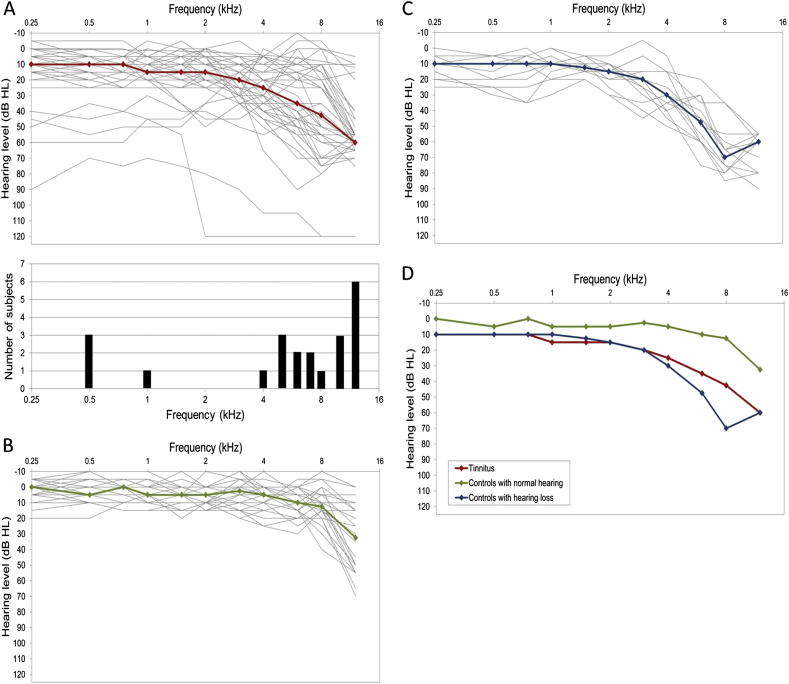
Individual (grey lines) and median (coloured lines) audiometric thresholds for 3 groups of participants: A: tinnitus, B: controls with normal hearing, C: controls with hearing loss, D: superposition of the medians from panels A–C. Additionally, bottom panel A shows the distribution of dominant tinnitus pitch derived from the similarity ratings.

There were two control groups. One comprised 7 people with hearing loss (‘hearing loss’ controls; PTA(0.25–8 kHz) > 20; mean age 64 years 7 months), while the other comprised 13 people with normal hearing (‘normal hearing’ controls; mean age 42 years 9 months); where normal hearing was defined as PTA(0.25–8 kHz)≤20. For the individual hearing profiles see Fig. 1. None of the ‘normal hearing’ participants reported any day to day hearing problems.

### 2.2 Tone stimuli

Auditory stimuli were 300-ms tone bursts presented at 40 dB SL to adjust for the degree of individual hearing loss at each particular frequency. Frequency conditions were defined according to the individual audiogram for each ear and tinnitus spectrum for the prevailing tinnitus sound. Conditions were: (i) a ‘control’ tone in the region of normal hearing, (ii) a tone corresponding to the individual audiometric edge frequency, (iii) a tone corresponding to the dominant tinnitus pitch, and (iv) a tone within the area of hearing loss. The audiometric edge was determined objectively using the ‘broken stick’ procedure performed for log frequency, as previously described in Sereda et al. (2011).

For the tinnitus group, the geometric mean of frequency of the selected tones was as follows: (i) control = 1.2 kHz, (ii) edge = 3.1 kHz, (iii) tinnitus pitch = 3.1 kHz. It was not possible in all cases to easily determine tones in those conditions. For some tinnitus participants, 1 kHz corresponded to the audiometric edge (*N* = 3) or dominant tinnitus pitch (*N* = 2). In those cases, the chosen control tone was instead a 0.5 kHz tone. For the one participant with low-frequency hearing loss, an 8-kHz tone, which was within normal hearing range, was selected as a control tone. For ten tinnitus participants, their hearing loss was sufficiently severe that the tinnitus tone could not be presented at 40 dB SL within the output capabilities of the sound delivery system. In those cases, an alternative tone was selected that was near to the tinnitus tone and within the region of hearing loss, but could be presented at 40 dB SL. That frequency was categorised separately as (iv) a hearing loss tone (mean = 4.6 kHz). For two individuals with asymmetric hearing, tones in condition (iv) were presented to the worse ear at maximum output. However, the corresponding evoked responses were not included in the analysis because these tones did not reach 40 dB SL. Table 2 reports the individual participants' dominant tinnitus pitch, hearing loss edge frequency and the frequencies presented in the MEG study that were included in the analysis.

Condition (iii) corresponding to the tinnitus pitch was not applicable for control groups. For the hearing-loss controls, the geometric mean of the frequencies of the selected tones was as follows: (i) control = 1.1 kHz, (ii) edge = 2.4 kHz, and (iv) hearing loss = 4.8 kHz. Normal-hearing controls were presented with tones corresponding to 1.0, 4.0 and 8.0 kHz and for the purpose of analysis, these were all considered to be control tones. Geometric means of the frequencies presented in the MEG across the three groups of participants are shown in Table 3.

Tone stimuli were generated in advance and presented using Presentation software (Neurobehavioural Systems, Albany, California, USA) and attenuated using TDT PA5 attenuators (Alachua, Florida, USA). The maximum sound level output for any tone frequency was limited to 100 dB SPL. Tones were delivered to left and right ears using Etymotic transducers (Etymotic Research – ER2) inside the magnetically shielded room. To protect the MEG sensors from potential magnetic interference due to the proximity of the transducers to the MEG helmet, each transducer was placed in specially designed mu-metal casing for shielding.

### 2.3 Neuromagnetic recordings and analysis

MEG data were recorded with participants in a supine position and in a magnetically shielded room using a whole cortex 275-channel CTF MEG system (VSM Medtech, Port Coquitlam, British Columbia, Canada) equipped with 3rd order gradiometer sensor configuration. A sampling rate of 600 Hz was used, applying a 150-Hz low-pass anti-aliasing hardware filter. The MEG data was collected in trials of 1 s duration which included a 100 ms prestimulus baseline. Each tone condition was repeated 120 times delivered in a random sequence to each ear with a varying inter-stimulus interval between 1.5 and 3.0 s. Participants were instructed to listen attentively without performing any task. To minimise contamination of the evoked potentials with alpha rhythm, subjects were asked to keep their eyes opened and focus on a set point. The experiment lasted about 25 min.

During data acquisition, the position of the participants' head inside the scanner was monitored by placing three coils at the nasion and the left and right pre-auricular fiducial points. A head motion tolerance of 5 mm was applied; none of the datasets violated this limitation. Following the data collection, the position of the coils and the head shape on the scalp surface were recorded using a 3D digitiser (Polhemus isotrack, Colchester, Vermont, USA).

**Fig. 2:**
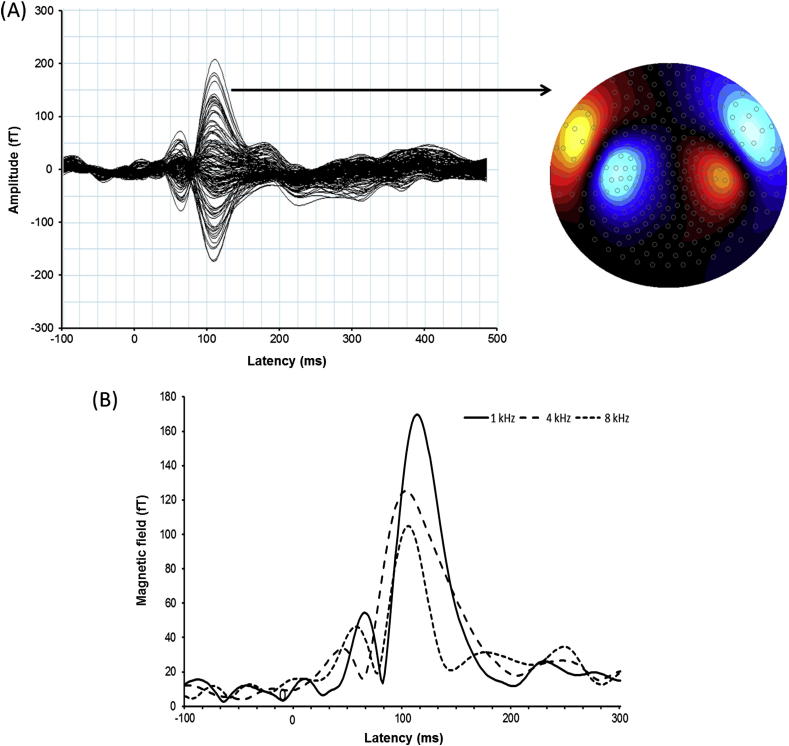
(A) An overlay of auditory evoked field recorded from 275 channels from a representative control participant with normal hearing to a 1 kHz tone presented to the right ear. A clear N1m can be seen at 110 ms after stimulus onset (0 ms). The distribution of the magnetic field over the left hemisphere shows clear dipolar pattern in both left and right hemispheres (right panel). (B) Auditory evoked responses (RMS) for the same participant in response to 1, 4 and 8 kHz stimulation. The decrease in N1m amplitude as a function of frequency is particularly evident in this plot.

The dataset was mean corrected and a 3rd order synthetic gradiometer was applied. Trials with artefacts were excluded from the analysis following visual inspection. The data was then filtered in the 1–20 Hz band and separately averaged for each tone condition to determine contra- and ipsi-lateral N1m responses in each participant. The N1m response was identified by visual inspection as a dipole pattern distributed over the temporal region with a peak in the time window between about 80 and 190 m after sound onset. Ten channels with the largest amplitude responses (5 sinks and 5 sources) in each hemisphere were identified and chosen for further analysis. For each hemisphere, the root mean square (RMS) of the field strength was calculated for each sample timepoint, averaged across the chosen channels (see Chait et al., 2004). The peak N1m amplitude and peak latency was derived from the RMS time course (Fig. 2). N1m amplitude was taken as the maximum RMS value of the averaged response. The latency of N1m was the time to the peak RMS value.

## 3 Results

A linear mixed effects analysis of variance (ANOVA) was performed using PASW Statistics 17 which enabled within- and between-subject comparisons. This was performed separately for both peak N1m amplitude and latency. We tested the main effect of group (tinnitus, hearing loss controls, normally hearing controls), tone category (control, edge, tinnitus pitch, hearing loss), laterality (contralateral, ipsilateral) and ear of presentation (left, right). In order to adjust for frequency differences between participants within each tone category log frequency was included in the analysis as a linear covariate. The logarithmic transformation was applied to allow for the non-linear relationship between frequency and the response. We also tested all interactions of main variables and covariate with group (group x tone category, group x laterality, group x ear, group x frequency) as well as tone category x laterality.

**Fig. 3:**
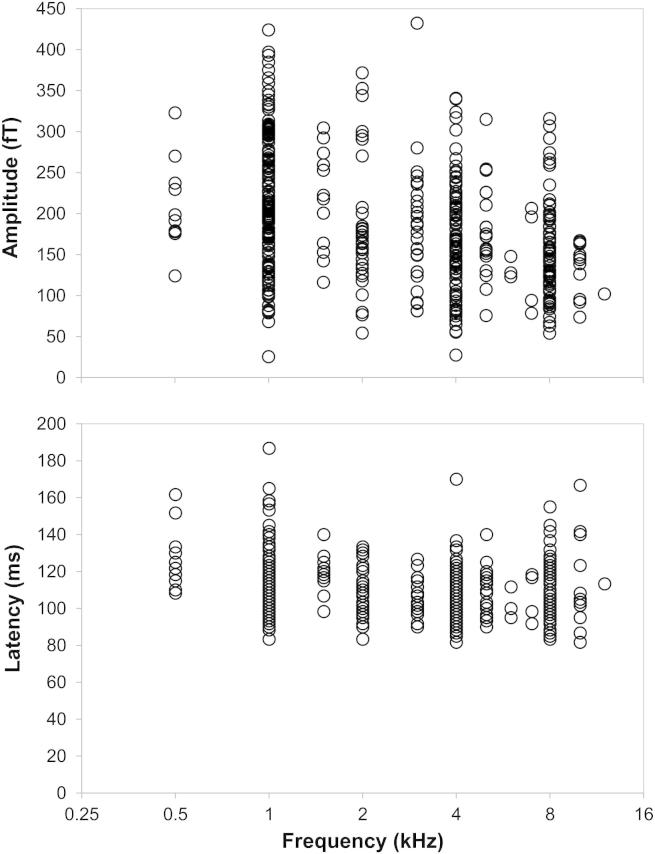
Amplitudes and latencies of N1m responses to all tone categories in all participants as a function of frequency.

For the covariate, N1m amplitude and latency significantly decreased as a function of frequency (F[1, 413.51] = 6.52, *p* = 0.011 and F[1, 383.49] = 7.69, *p* = 0.006, respectively). However, the data plotted in Fig. 3 indicate that this relationship was much more pronounced for amplitude than for latency. For example, from 1 to 10 kHz along the frequency axis, there was a 37.1% reduction in mean amplitude (78.5 fT), but only a 0.9% difference in latency (1 ms). This result seems to be in agreement with the majority of previous studies showing decreasing N1m amplitudes with increasing frequency and the relative lack of frequency dependence of N1m latency to tones of 1 kHz and above (Gabriel et al., 2004; Fujioka et al., 2002; Naka et al., 1999). The interaction between frequency and group was not significant for amplitude (F[2, 405.29] = 2.10; *p* = 0.12), indicating that both tinnitus participants and controls showed an equivalent pattern of frequency dependence. Although latency for hearing-loss controls decreased as a function of frequency more rapidly than the other two groups (F[2, 395.17] = 3.58; *p* < 0.029), absolute changes in latency were very small.

**Fig. 4:**
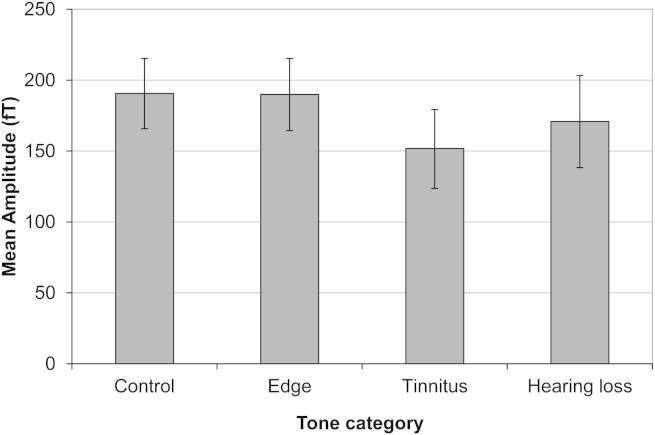
Mean amplitudes of N1m for four categories of tones (control, edge frequency, tinnitus frequency, frequency within area of hearing loss) corrected for frequency. Error bars represent the 95% confidence intervals for each tone category.

**Fig. 5:**
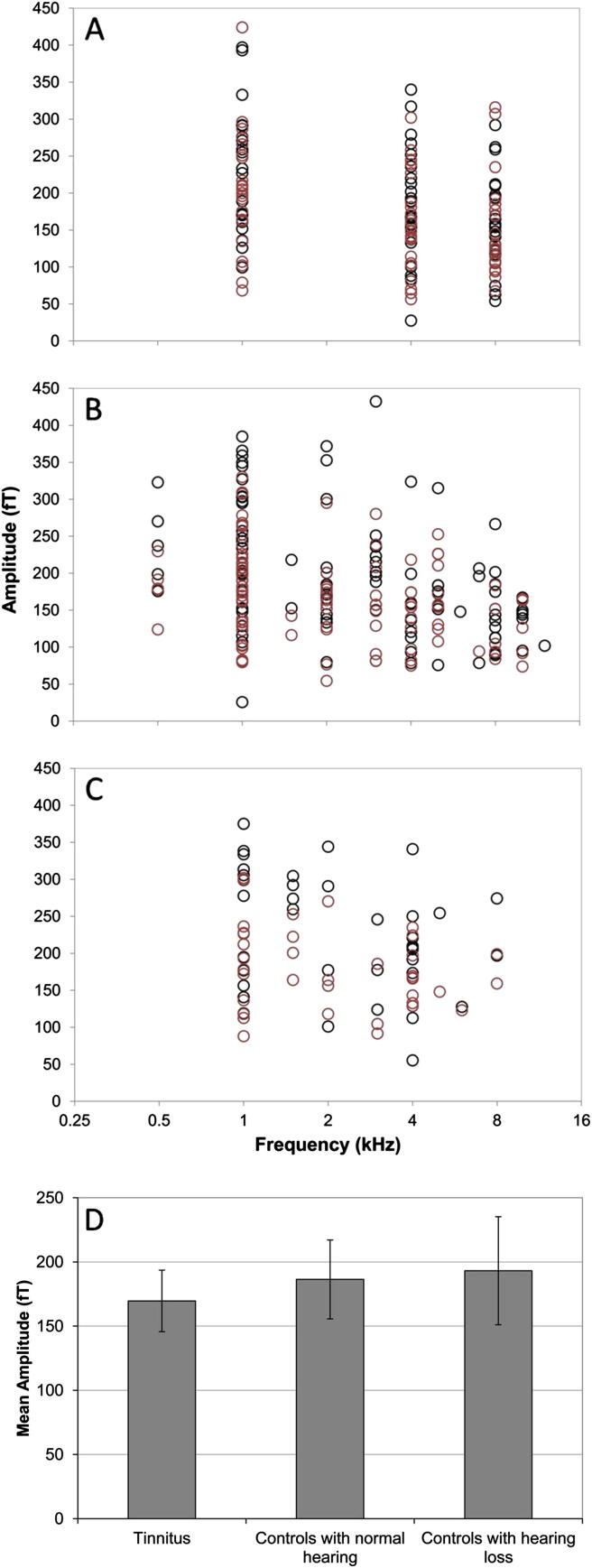
Individual (A, B, C) and mean (D) amplitudes of N1m for three groups of participants (controls with normal hearing (A), tinnitus (B) and controls with hearing loss (C)) corrected for frequency. Contralateral responses are shown by black and ipsilateral by red circles. Error bars represent 95% confidence intervals for each tone category.

Having accounted for the effect of frequency in the model, our results indicate a significant residual effect of tone category on N1m amplitude (F[3, 394.48] = 3.35, *p* = 0.019). Overall, the responses to the frequency corresponding to the dominant tinnitus pitch were smaller than for control and edge frequencies (*p* = 0.013) (Fig. 4). However, we did not find significant differences between amplitudes of the N1m responses between three groups of participants (F[2, 71.89] = 0.17; *p* = 0.85; Fig. 5). There was no significant effect of tone category on N1m latency (F[3, 394.14] = 0.20; *p* = 0.89), nor group on N1m latency (F[2, 102.39] = 1.49; *p* = 0.23). We infer that the overall pattern of N1m responses for the tinnitus group did not differ from the controls as there was no interaction between group and tone category for amplitude (F[2, 395.89] = 0.96; *p* = 0.38) and latency (F[2, 387.26] = 2.56; *p* = 0.08). In general, we therefore found no evidence of tinnitus-specific changes in the pattern of N1m responses.

For the main effect of laterality, N1m amplitude was greater in the contralateral hemisphere (F[1, 375.51] = 27.85, *p* = 0.001), with shorter latency (F[1, 378.15] = 52.78, *p* = 0.001). This pattern was found regardless of the group, tone category and ear of presentation. Contralateral dominance reflects the cross-over organisation of the ascending auditory pathway and our result concurs with previous human neuroimaging studies in this regard (e.g., Näätänen and Picton, 1987; Kanno et al., 1996; Langers et al., 2005).

As for the ear of presentation, there was no effect on amplitude (F[1, 378.76] = 0.04; *p* = 0.84), but N1m latency was significantly shorter when stimuli were presented to the left than to the right ear (F[1, 384.81] = 5.14, *p* = 0.024). As in the case for frequency, the magnitude of the latency difference across ears was so small as to be not functionally meaningful (3.4 ms).

To further discriminate the effects of hearing loss from the effects of tinnitus on N1m amplitudes we compared tinnitus participants with hearing loss with controls with hearing loss. Mixed effects ANOVA testing all the effects assessed in the main analysis was performed. There was no difference between amplitudes of N1m between tinnitus with hearing loss participants and controls with hearing loss (F[1, 52.17] = 0.14, *p* = 0.71). However, similarly to the results of the main analysis we found significant effects of tone category on amplitudes of N1m, where amplitudes for the tinnitus pitch were lower than for control (*p* = 0.031) and edge frequency (*p* = 0.015) and similar to the hearing loss frequency (*p* = 0.28).

To further explore the effects of tinnitus laterality (unilateral vs bilateral) and ear (tinnitus vs non-tinnitus ear) on amplitudes of N1m responses additional linear mixed effects ANOVA was performed. We tested the main effect of group (unilateral vs bilateral tinnitus), tone category (control, edge, tinnitus pitch, hearing loss), laterality (contralateral, ipsilateral) and ear of presentation (tinnitus, non-tinnitus) with log frequency included in the analysis as a linear covariate. We did not find differences in amplitudes of N1m responses between unilateral and bilateral tinnitus patients (F[1, 41.54] = 0.09, *p* = 0.77) nor between tinnitus and non-tinnitus ear (F[1, 164.46] = 0.68, *p* = 0.41). Similar to the main analysis, we found a significant effect of tone category (F[3, 170.99] = 5.98, *p* = 0.001) and frequency (F[1, 176.68] = 39.21, *p* = 0.001) on N1m amplitudes.

## 4 Discussion

The present study set out to characterise the auditory N1m response elicited by tones of different frequencies (control tone, edge frequency, dominant tinnitus pitch, hearing loss frequency) in tinnitus participants and to compare them with two non-tinnitus control groups; normal hearing subjects and with subjects with hearing loss. In general, we predicted tinnitus-related differences most likely in the N1m amplitude for tone frequencies corresponding to the dominant tinnitus pitch and/or the audiometric edge frequencies. Our results have shown that amplitudes of N1m response indeed depended on the tone category, even after controlling for frequency, but crucially there was no difference in this pattern across tinnitus and control groups. In general, the N1m for the dominant tinnitus pitch was significantly smaller than for the control and audiometric edge frequencies and was similar to the tone within the hearing-loss region. Therefore we postulate that differences in evoked responses pattern across tone category seem more related to the hearing loss than to the presence of tinnitus.

### 4.1 Effect of frequency on the N1m

In agreement with the previous literature we found more pronounced frequency dependent changes for N1m amplitude than for latency (Naka et al., 1999; Fujioka et al., 2002; Gabriel et al., 2004). The amplitude of N1m decreased as stimulus frequency increased. The shift in latency was minimal (only 1 ms between 1 and 10 kHz), therefore negligible. Possible explanations of the decrease in N1m amplitude with frequency include a decrease in the number of phase-locked neurons for higher frequencies relative to lower frequencies in such a way that rate coding declines to play a role and that place coding begins to dominate (Gabriel et al., 2004). Another possibility could be due to the tonotopic layout of the auditory fields with respect to the surface of the scalp where areas of the map responding to high frequencies lie deeper than those responding to low frequencies (see Fujioka et al., 2002). A number of studies cross-validating the source of the N1 with data from other neuroimaging modalities indicate that this evoked component is likely to be generated from different sources in the intermediate and lateral parts of Heschl's gyrus and in areas posterior to it (e.g., Godey et al., 2001; Mayhew et al., 2010). Functional MRI studies of tonotopic mapping across human auditory cortex confirm that, within these regions, low-frequency response foci predominate (e.g., Langers and van Dijk, 2011; Talavage et al., 2004).

### 4.2 Effect of hearing loss on evoked responses

As reviewed in the introduction, many of the current models of tinnitus generation attribute the tinnitus sensation to altered neuronal activity within the central auditory system triggered by damage to the auditory periphery. Depending on the precise details of the proposed neural mechanism, we might expect differences in the amplitude and/or latency of the evoked responses for tones corresponding to the edge frequency of the hearing loss and/or to the dominant tinnitus pitch compared to a control tone, and for this to be different in the tinnitus group compared to controls.

Enhancement of the N1m amplitude for a tone corresponding to the audiometric edge frequency might be directly predicted by those models which consider tinnitus to be the consequence of a shift in the frequency tuning properties of neurons above the audiometric edge towards those of lower frequencies (Robertson and Irvine, 1989; Harrison et al., 1991; Rajan et al., 1993; Eggermont and Komiya, 2000). Similar effects are predicted by those authors who attribute tinnitus to contrast enhancement of neural activity spanning the audiometric edge (e.g. Kiang et al., 1969; Llinas et al., 2005). In general, lateral-inhibition models of tinnitus produce a ‘tinnitus’ activity peak at a discontinuity or edge of the profile of spontaneous activity along the tonotopic axis (Gerken, 1996). This model would therefore predict the modification of evoked response to edge and/or tinnitus frequency. Our data are difficult to consolidate with these views. We observed the pattern of responses for the edge frequency to be similar to that for the control frequency, regardless of the group tested. It is worth noting that those models also postulate that the dominant tinnitus pitch should be located at the edge of hearing loss (Eggermont and Roberts, 2004; Schaette and Kempter, 2009). However, recent data have contradicted this by finding a less specific relationship to the area of hearing loss (Pan et al., 2009; Sereda et al., 2011).

Enhancement of the N1m amplitude for a tone corresponding to the area of hearing loss would be consistent with the view that tinnitus is generated by synchronous spontaneous activity which develops among hyperactive neurons in the deafferented region (Eggermont and Roberts, 2004). Again, our data do not support this view as we observed reduction of the amplitude of N1m response for both the tinnitus pitch and tone within the area of hearing loss.

No change in the N1m amplitude would be consistent with homeostatic plasticity models that postulate tinnitus to be a side effect of plasticity mechanisms that normally ensure proper function of the auditory brain through an elevation of central spontaneous activity in the frequency range that is affected by hearing loss (Schaette and Kempter, 2006, 2008, 2009). Although it is hard to predict how such changes in activity would affect evoked responses on the cortical level, the data reported by Schaette and McAlpine (2011) are suggestive of a renormalisation of reduced evoked responses occurring somewhere between auditory nerve and midbrain nuclei. In the light of these findings we might expect no difference in the amplitudes of cortical evoked responses between participants with tinnitus and controls. As above, our data do not support normalisation of the responses at the level of auditory cortex as we found reduced N1m amplitudes for the tones within the area of hearing loss.

Among our tinnitus group, we had five patients with normal hearing thresholds and it may be argued that these should form a separate experimental group. However, four of these had various degrees of hearing loss for frequencies above 8 kHz. In the context of tinnitus, these cannot be considered as normally hearing. This is because at least in theory, hearing loss at any frequency can be related to tinnitus. Moreover, recently Schaette and McAlpine (2011) have shown that patients with normal clinical audiograms may have “hidden hearing loss” possibly caused by damage to auditory nerve fibres.

The fact that the frequency corresponding to the dominant tinnitus pitch typically lies within the area of hearing loss (Henry and Meikle, 1999; Noreña et al., 2002; Pan et al., 2009; Sereda et al., 2011) is a major consideration for the interpretation of the results. We were able to measure the evoked response to a tone matching the dominant tinnitus pitch for only twelve people with tinnitus due to the output restrictions of the sound delivery system. There was no difference between N1m amplitudes for the tinnitus pitch and hearing loss frequency. While this may be unsurprising, because the frequency of the tone in each category was equivalent (see Table 2), this non-significant effect at least points to the suggestion that there is nothing special about the N1m response corresponding to the tinnitus frequency. Our data do not provide any evidence that MEG can measure correlates of tinnitus-related cortical reorganisation of frequency coding. Rather, the generalised reductions in N1m amplitude for the high-frequency (>5 kHz) tones implicate hearing loss as a more likely causal factor.

## 5 Conclusions

In line with recommendations in the literature, the current study controlled for comorbid symptoms such as hyperacusis and compared tinnitus participants to a hearing loss matched control group. We did not find any tinnitus specific changes in the pattern of N1m responses. The reduction of amplitudes of N1m for the tinnitus frequency and lack of difference between amplitudes for tinnitus frequency in comparison to the frequency within the area of hearing loss suggests that the differences in the evoked responses pattern in tinnitus participants point to the hearing loss rather than tinnitus itself as a causal factor.

## Figures and Tables

**Table 1:** Characteristics of 22 participants with tinnitus and frequencies tested in MEG that were included in the analysis. ‘TI’ refers to tinnitus and ‘HL’ refers to hearing loss.

Participant	Dominant TI pitch (kHz)	Edge frequency (kHz)		Tones tested (kHz)							
		Left	Right	Left				Right			
				Control	Edge	TI	HL	Control	Edge	TI	HL
1	5	x	x	1; 3	x	5	x	1; 3	x	5	x
2	10	8	8	1	8	10	x	1	8	10	x
3	12	8	8	1	8	x	10	1	8	x	10
4	0.5	8	8	1	8	0.5	x	1	8	0.5	x
5	10	5	5	1	5	10	x	1	5	10	x
6	12	2	3	1	2	x	8	1	3	x	8
7	12	3	4	1	3	x	4	1	4	x	7
8	4	3	6	1	3	4	x	1	6	4	x
9	1	4	4	8	4	1	x	8	4	1	x
10	6	2	3	1	2	x	3	1	3	x	x
11	10	2	3	1	2	x	5	1	3	x	5
12	12	4	4	1	4	12	x	x	x	x	x
13	7	3	3	1	3	7	x	1	3	7	x
14	5	2	2	1	2	5	x	1	2	5	x
15	12	8	x	1	5	x	8	x	x	x	x
16	12	x	1.5	x	x	x	x	1	1.5	x	4
17	0.5	1	1	x	1	0.5	4	x	1	0.5	4
18	0.5	2	5	1	2	0.5	x	1	5	0.5	x
19	8	2	1.5	1	2	x	4	1	1.5	8	x
20	5	2	2	1	2	x	x	1	2	x	x
21	6	x	1	x	x	x	1; 2	x	1	x	2
22	7	3	2	1	3	x	x	1	2	x	x
Mean *(SD)*	5.2 (*3.0*)	3.2 (*1.8*)	3.1 (*1.9*)								

**Table 2:** Characteristics of the three groups of participants. ‘TI’ refers to tinnitus.

		TI	‘Hearing loss’ controls	‘Normal hearing’ controls
Gender	Male	11	3	5
	Female	11	4	8
Age	Mean *(SD)*	55.7 *(13.1)*	62.1 *(7.9)*	40.6 *(14.2)*
Pure-tone average (0.25 - 8 kHz)	Mean Left *(SD)*	23.8 *(17.7)*	25.8 *(5.5)*	6.2 *(4.9)*
	Mean Right *(SD)*	21.9 *(17.3)*	22.2 *(6.0)*	4.8 *(4.4)*
TI duration (years)	Mean *(SD)*	9.5 *(10.6)*	NA	NA
TI handicap inventory (THI)	Mean *(SD)*	39.4 *(21.2)*	NA	NA
TI quality	Tonal	16	NA	NA
	Hissing	2	NA	NA
	Ringing	4	NA	NA
TI laterality	Left	5	NA	NA
	Right	6	NA	NA
	Bilateral	11	NA	NA
Hyperacusis score	Mean *(SD)*	13.8 *(7.1)*	0	0

**Table 3:** Geometric mean and geometric standard deviation of frequencies presented in the MEG across three groups of participants.

		Average frequency across group (kHz)			
		Control	Edge	Tinnitus pitch	Hearing loss
Tinnitus	Mean *(SD)*	1.2 *(1.7)*	3.1 *(1.8)*	3.1 *(3.3)*	4.7 *(1.9)*
‘Hearing loss’ controls	Mean *(SD)*	1.1 *(1.2)*	2.4 *(1.5)*	NA	4.8 *(1.3)*
‘Normal hearing’ controls	Mean *(SD)*	1, 4, 8	NA	NA	NA
